# Apparent diffusion coefficient for assessing Crohn’s disease activity: a meta-analysis

**DOI:** 10.1007/s00330-022-09149-9

**Published:** 2022-09-28

**Authors:** Maximilian Thormann, Bohdan Melekh, Caroline Bär, Maciej Pech, Jazan Omari, Andreas Wienke, Hans-Jonas Meyer, Alexey Surov

**Affiliations:** 1 Department of Radiology and Nuclear Medicine, University of Magdeburg, Leipziger Str. 44, 39120 Magdeburg, Germany; 2 Institute of Medical Epidemiology, Biostatistics, and Informatics, Martin-Luther-University Halle-Wittenberg, Halle, Germany; 3 Department of Diagnostic and Interventional Radiology, University of Leipzig, Leipzig, Germany

**Keywords:** Crohn’s disease, Magnetic resonance imaging, Diffusion-weighted imaging, Meta-analysis

## Abstract

**Purpose:**

To analyze relationships betweenapparent diffusion coefficient (ADC) and activity parameters of Crohn’s disease, e.g., length and wall thickness, CRP, FCP, MaRIA, CDAI, SES-CD, histologic inflammatory activity score, and the histological fibrotic score, based upon published data.

**Materials and methods:**

MEDLINE library, Scopus, and Embase databases were screened for association between ADC and activity parameters of Crohn’s disease in patients with Crohn’s disease up to Mai 2021. Overall, 21 studies with 1053 patients were identified. The following data were extracted from the literature: number of patients, correlation coefficients between ADC and length as well as wall thickness, CRP, FCP, MaRIA, CDAI, and SES-CD, inflammatory activity score, and fibrotic score. Associations between ADC and activity parameters were analyzed by Spearman’s correlation coefficient. The studies’ methodologic quality was evaluated by using the Quality Assessment of Diagnostic Studies (QUADAS 2) instrument, revealing a low risk of bias.

**Results:**

In the overall sample, the pooled correlation coefficient between ADC and CDAI was −0.8 (95% CI = [−0.94; −0.65]), between ADC and MaRIA −0.66 (95% CI = [−0.79; −0.53]). A strong association was observed between ADC and SES-CD with a pooled correlation of −0.66 (95% CI = [−0.87; −0.46]). The pooled sensitivity to discriminate between involved and non-involved bowel segments was 0.89, with an area under the curve of 0.89

**Conclusions:**

ADC showed strong inverse correlations with CDAI, MaRIA, and SES-CD scores. However, the role of ADC in assessing fibrotic changes in the bowel wall is limited. ADC can reflect acute inflammatory reactions but not systemic inflammation.

**Key Points:**

• *ADC value can reflect acute inflammatory reactions but not systemic inflammation.*

• *ADC is inversely correlated with CDAI, MaRIA, and SES-CD.*

• *The role of ADC in assessing fibrotic changes in the bowel wall is limited.*

## Introduction

Crohn’s disease (CD) is one of the common chronic disorders in the industrialized world with an incidence of 3–20 cases per 100,000 and a wide spectrum of clinical manifestations [1–3]. Due to the varying pattern of CD, disease activity must be closely monitored. Severity of disease can be determined with quantitative or semiquantitative assessment of inflammation in the bowel [4].

Aside from laboratory, endoscopic, and enterographic examinations, magnetic resonance enterography (MRE) is the most important imaging modality for monitoring disease activity. It allows non-invasive investigation of the gastrointestinal tract and provides an assessment of inflammatory activity and potential complications in all bowel segments [5–9]. Current MR protocols include rapid MR sequences for data acquisition during a single breath-hold with minimal motion artefacts and rapid morphological sequences with a gadolinium-chelate-enhanced series [10]. However, with rising concerns about gadolinium retention in different organs, particularly the brain, the repeated application of gadolinium-based contrast agents is viewed critically [11, 12]. Therefore, alternative non-enhanced methods for repeated lifelong disease monitoring have gained relevance. MRI protocols usually include diffusion-weighted sequences (DWI-MRE), allowing for qualitative and quantitative assessment of random motion of water molecules in biological tissues. The use of DWI is recommended as an optional sequence for Crohn’s disease by the European Society of Gastrointestinal and Abdominal Radiology in the latest consensus statements [13]. DWI could therefore potentially replace the contrast-enhanced sequences with comparable diagnostic power [6].

Choi et al [14] showed in a meta-analysis that accuracy and diagnostic strength of DWI-MRE in assessing bowel inflammation were overestimated in some studies. The correlation of DWI-derived apparent diffusion coefficient (ADC) with disease activity produced heterogeneous results and clinical relevance of quantitative ADC measurements could not be established due to the limited number of studies available at the time. With a growing interest in DWI-MRE and ADC in CD patients in recent years, this paucity has been largely cleared. A recent meta-analysis involving nine studies with pediatric patients with inflammatory bowel disease reported a sensitivity and specificity of DWI-MRE of 0.93 and 0.95, respectively [15]. In the meta-analysis by Choi et al [14], the data was based mostly on studies explaining the diagnostic value of DWI images and not the quantitative ADC value. Moreover, the published data has been increasingly growing since then, necessitating an updated analysis. To our knowledge, no systematic evaluation of the associations of ADC values with inflammation and fibrosis scores in CD has been performed in an adult population [16–29].

The aim of the present meta-analysis was to analyze the role of ADC in assessing disease activity in patients with CD.

## Methods

The Preferred Reporting Items for Systematic Reviews and Meta-Analyses statement (PRISMA) was used for the literature search [30].

### Literature search

MEDLINE library, Scopus, and Embase online databases were checked to identify studies for associations between ADC and different activity parameters of Crohn’s disease up to Mai 2021 using the following search criteria: “(Crohn) OR (inflammatory bowel disease) OR (IBD) AND (DWI) OR (diffusion weighted imaging) OR (ADC) OR (apparent diffusion coefficient).” Only papers written in English were included.

### Inclusion criteria

The first primary endpoint of the meta-analysis was the reported correlation between quantitative ADC measurements and activity parameters of Crohn’s disease. The second primary endpoint was the reported diagnostic abilities of ADC values for discrimination purposes of acute inflammation and fibrosis.

Studies (or subsets of studies) were included if they satisfied the following criteria:

1. Patients with Crohn’s disease (based on standard clinical, endoscopic, imaging, and histologic criteria);
2. Patients, who underwent MR enterography with DWI sequence quantified by ADC values;
3. Correlation coefficient between ADC and activity parameters of Crohn’s disease;
4. Receiver operating characteristic analysis with reported sensitivity, specificity, and area under the curve (AUC) for the discrimination analysis between involved and non-involved bowel segments.

### Exclusion criteria

Exclusion criteria were as follows:

1. Systematic review;
2. Case reports;
3. Conference abstracts, letter, editorials, meta-analysis, guidelines;
4. Non-English language;
5. Studies that analyzed patients with colitis ulcerosa or colitis ulcerosa and Crohn’s disease together.

Two readers (A.S. with 18 years of experience in radiology and B.M. with 9 years of experience in radiology) independently evaluated all articles and studies. In cases of disagreement, a third observer (H.J.M. with 6 years of experience) was consulted to reach a decision in consensus.

### Data extraction and quality assessment

Information was extracted on study characteristics (authors, year of publication, study design), demographic and clinical characteristics (sample size, male to female ratio, patient age), activity parameters, and correlation coefficients between ADC and activity of Crohn’s disease. The activity parameters included wall thickness and length, fecal calprotectin (FCP) and C-reactive protein (CRP), a magnetic resonance index of activity (MaRIA) and Crohn’s disease activity index (CDAI), endoscopic activity score (SES-CD), histologic inflammatory activity score, and also the histological fibrosis score.

**Fig. 1:**
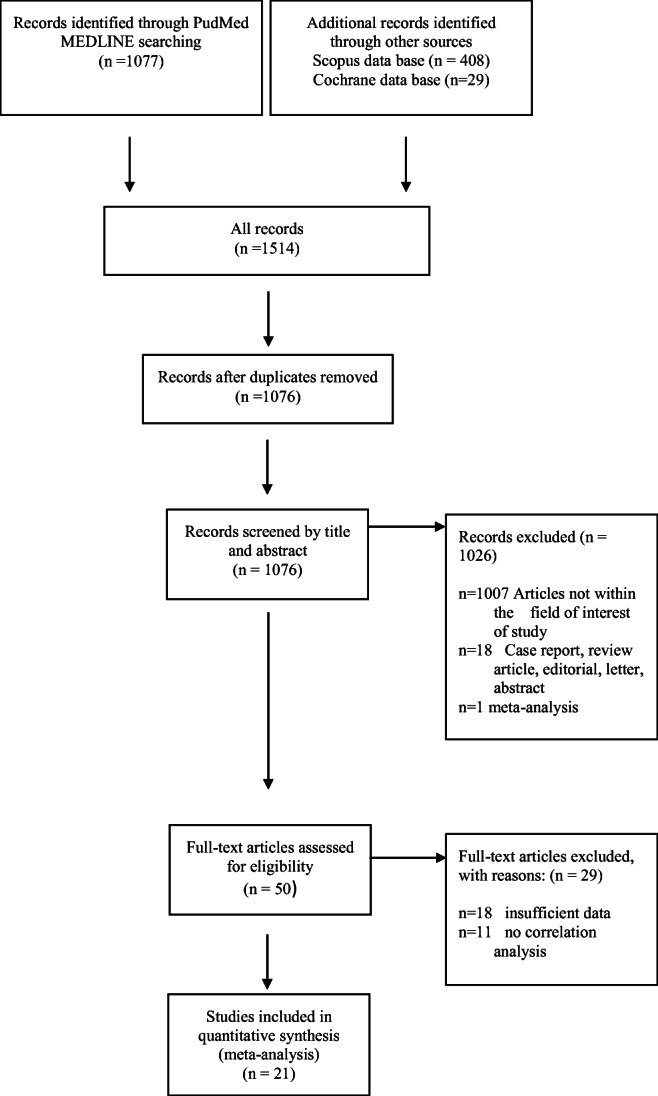
Flowchart of the data acquisition

In accordance with a wide spectrum of different activity parameters of Crohn’s disease, we divided all data into subgroups for assessing the correlation with ADC: (1) studies with an investigated correlation between ADC and morphological changes such as length and wall thickness; (2) ADC and laboratory parameters such as FCP and CRP; (3) ADC and activity indices: MaRIA and CDAI; and (4) ADC and SES-CD, histologic inflammatory activity score, and the histological fibrosis score (Fig. 1).

**Fig. 2:**
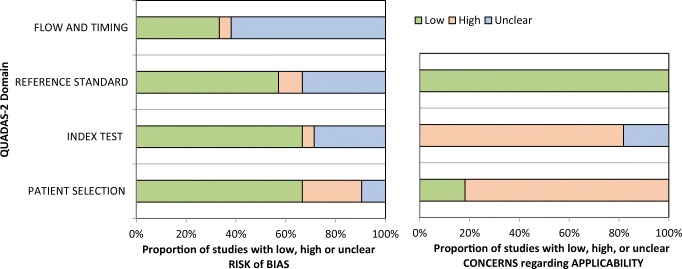
QUADAS-2 quality assessment of the included studies. Most studies showed an overall low potential for sources of bias

For the present meta-analysis, our search criteria identified 1514 articles. Duplicate records, review articles, case reports, meta-analyses, non-English publications, and articles which were not within the field of interest were excluded (*n* = 1464) (Fig. 2). As a next step, full-text reviews of the remaining papers (*n* = 50) were performed. Thereafter, 26 articles were excluded, because they were not in the field of interest and did not contain an analysis of the correlation between ADC and Crohn’s disease activity. Therefore, a total of 21 studies were involved in the analysis (Fig. 1) [16, 18–29, 31–38].

The methodologic quality of the studies was evaluated by using the Quality Assessment of Diagnostic Studies (QUADAS 2) instrument [39]. The following parameters were assessed for low, moderate, or high risk of bias: flow and timing, reference standard, index test, and patient selection.

### Data synthesis and analysis

The correlations between ADC and activity parameters of Crohn’s disease were calculated by Spearman’s correlation coefficient. The reported Pearson’s correlation coefficient was recalculated into Spearman’s correlation coefficients according to the previous description [40].

The statistical analysis of the meta-analysis was calculated in program RevMan 5.3 (computer program, version 5.3. Copenhagen: The Nordic Cochrane Centre, The Cochrane Collaboration, 2014). The heterogeneity was determined by using the inconsistency index *I*^2^ [41, 42] and defined as not important with a value of index between 0 and 40%; moderate—between 30 and 60%; substantial heterogeneity—50–90%; and finally considerable—more than 75% [43]. DerSimonian and Laird’s [44] random-effects models with inverse-variance weights were estimated without any further correction.

## Results

The 21 included studies comprised 1053 patients, of which 496 patients (47%) were female and 577 male (53%). There were 11 (52%) prospective and 10 (48%) retrospective studies. The size of the study population ranged from 20 to 229 patients with an average age of 26.5 years. Three studies reported results on pediatric patients [16, 37, 38], whereas the other studies only investigated adult study populations. Detailed characteristics of all studies are shown in Table 1.

**Table 1:** Characteristics of included studies

Authors	Year	Study design	Patients, *n*	Males:females	Age, mean	Tesla strength	Parameters
Abd-El Khalek Abd-Alrazek et al [25]	2018	Retrospective	72	40:32	30.9	1.5 T and 3 T	Wall thickness, MaRIA
Buisson et al [34]	2013	Prospective	31	11:20	26	1.5 T	MaRIA
Buisson et al [33]	2015	Prospective	44	21:23	27.9	1.5 T	SES-CD
Caruso et al [20]	2020	Retrospective	30	18:12	45.6	1.5 T	Histological inflammatory score, fibrosis score
Caruso et al [36]	2014	Retrospective	55	36:19	41	1.5 T	CRP, MaRIA, FCP, SES-CD
Cheng et al [27]	2019	Retrospective	51	37:14	29	3 T	SES-CD, MaRIA
Dillman et al [16]	2016	Prospective	28	17:11	15.3	3 T	Wall thickness, length, CRP, FCP
Du et al [21]	2021	Prospective	31	18:13	33	3 T	Histological inflammatory score, fibrosis score
Hectors et al [29]	2019	Prospective	27	18:9	42	1.5 T and 3 T	CRP, wall thickness, length, MaRIA
Klang et al [22]	2017	Retrospective	56	30:26	26	1.5 T	FCP, CRP
Li et al [23]	2017	Retrospective	43	27:16	26.8	3 T	SES-CD
Li et al [35]	2015	Prospective	47	29:18	27.9	3 T	CDAI
Li et al [28]	2019	Prospective	30	13:17	32.5	3T	Histological inflammatory score, fibrosis score
Li et al [24]	2018	Prospective	31	19:12	32.4	3 T	Fibrosis score
Neubauer et al [38]	2013	Retrospective	60	24:36	16	1.5 T	Wall thickness
Ream et al [37]	2013	Retrospective	46	23:23	14.3	1.5 T	Wall thickness, length
Strakšytė et al [18]	2020	Prospective	229	124:125	35.4	1.5 T	MaRIA
Tielbeek et al [32]	2014	Prospective	20	8:12	38	3 T	Fibrosis score
Wu et al [19]	2020	Retrospective	48	32:16	33.8	3 T	CDAI
Zhang et al [26]	2019	Prospective	24	14:10	30	3 T	Fibrosis score
Zhu et al [31]	2016	Prospective	50	18:32	32.3	3 T	CRP

The QUADAS-2 assessment revealed an overall low to moderate risk of bias in the included studies. The reference standard for inflammation differed between studies as some studies used histopathological assessment and some only clinical severity, which can result in bias. The patient selection can be considered relatively free from bias, as only three studies included pediatric patients with inherent differences from the adult population. Across studies, ADC values were measured as ADCmean values within a region of interest (ROI) of the inflamed bowel segment.

### Correlations between ADC and MR morphological changes

**Fig. 3:**
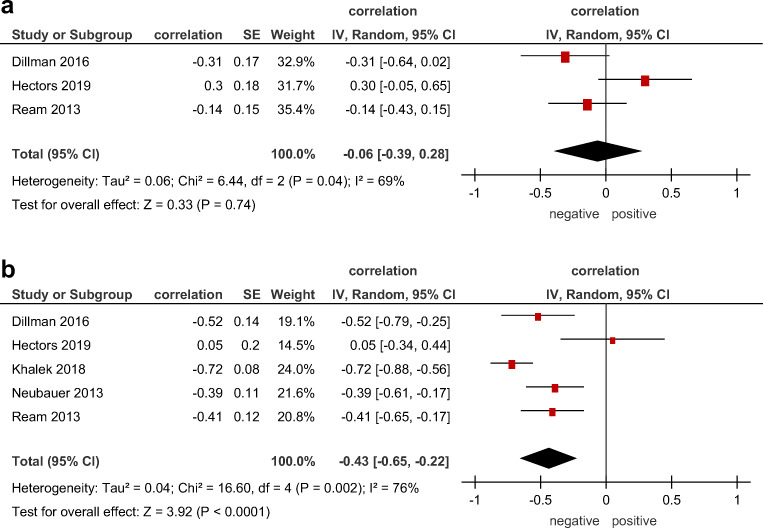
Forest plots of correlation coefficients between (**a**) ADC and length of inflamed bowel wall, (**b**) ADC and thickness of inflamed bowel wall

In 2 studies including 74 patients, data about relationships between ADC and extent of bowel affection were reported. The pooled correlation coefficient between these parameters was −0.06 (95% CI = [−0.39, 0.28]), *p* = 0.74, heterogeneity *τ*^2^ = 0.06 (*p* = 0.04), *I*^2^ = 69%, test for overall effect *Z* = 0.33 (Fig. 3a). Associations between wall thickness and ADC were analyzed in 5 studies comprising 233 patients. The pooled correlation coefficient was −0.43 (95% CI = [−0.65; −0.22]), *p* < 0.00001, heterogeneity *τ*^2^ = 0.04 (*p* = 0.002), *I*^2^ = 76%, test for overall effect *Z* = 3.92 (Fig. 3b).

### Correlation between ADC and disease activity scores

**Fig. 4:**
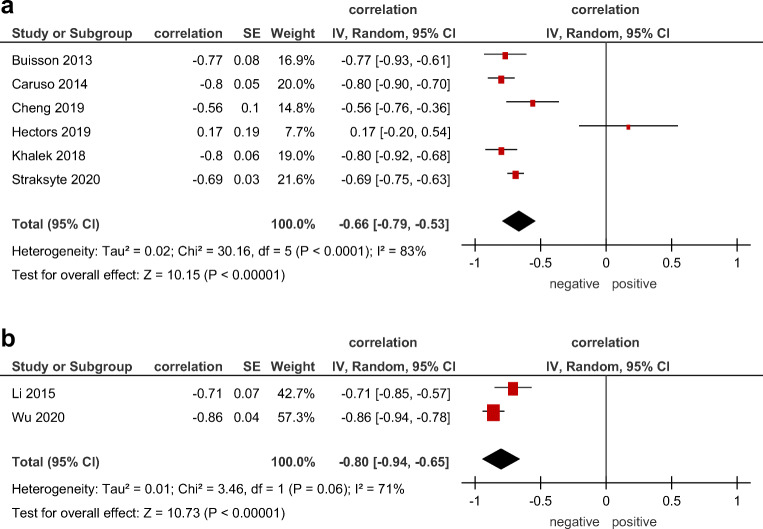
Forest plots of correlation coefficients between (**a**) ADC and magnetic resonance index of activity, (**b**) ADC and Crohn disease activity index

In 6 studies with 465 patients, a strong association between ADC and MaRIA was shown with a pooled correlation coefficient of −0.66 (95% CI = [−0.79; −0.53]), *p* < 0.000001, heterogeneity *τ*^2^ = 0.02 (*p* < 0.0001), *I*^2^ = 83%, test for overall effect *Z* = 10.15 (Fig. 4a).

Correlations between ADC and CDAI were reported in 2 studies (95 patients). The pooled correlation coefficient was −0.8 (95% CI = [−0.94; −0.65]), *p* < 0.000001, heterogeneity *τ*^2^ = 0.01 (*p* = 0.06), *I*^2^ = 71%, test for overall effect *Z* = 10.73 (Fig. 4b).

**Fig. 5:**
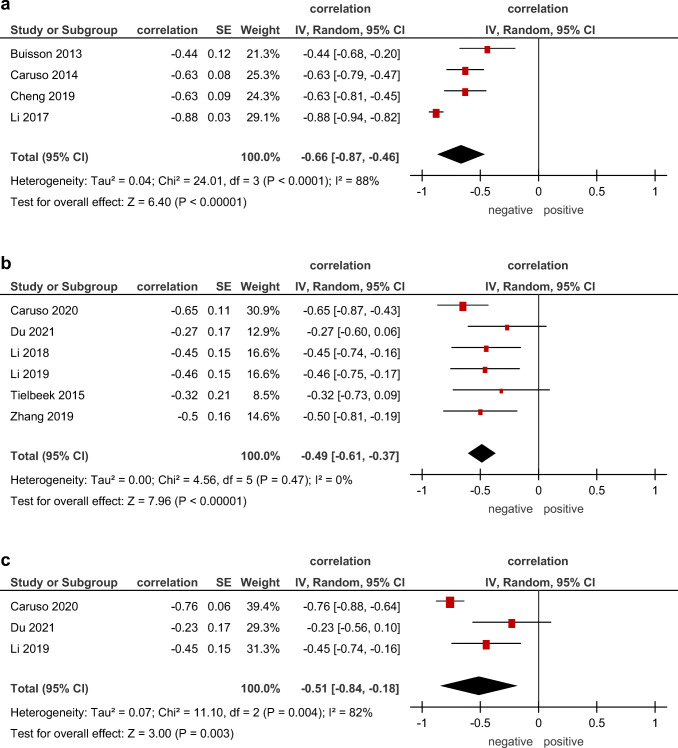
Forest plots of correlation coefficients between (**a**) ADC and endoscopic activity score, (**b**) ADC and histological fibrotic score, (**c**) ADC and histologic inflammatory score

In addition, correlations between ADC and morphological parameters, like SES-CD, histological fibrotic score, and histologic inflammatory score, were evaluated (Fig. 5a–c).

In 4 studies with 193 patients, associations between ADC and SES-CD were analyzed. The pooled correlation coefficient was −0.66 (95% CI = [−0.87; −0.46]), *p* < 0.000001, heterogeneity *τ*^2^ = 0.04 (*p* < 0.0001), *I*^2^ = 88%, test for overall effect *Z* = 6.40 (Fig. 5a).

Correlations between ADC and histological fibrotic score were reported in 6 studies (166 patients). The pooled correlation coefficient was 0.49 CI (95% CI = [−0.61; −0.37]), *p* < 0.000001, heterogeneity *τ*^2^ = 0.00 (*p* = 0.47), *I*^2^ = 0%, test for overall effect *Z* = 7.96 (Fig. 5b).

In 3 studies (91 patients), relationships between ADC and histologic inflammatory score were investigated. The pooled correlation coefficient was −0.51 (95% CI = [−0.84, −0.18]), *p* = 0.003, heterogeneity *τ*^2^ = 0.07 (*p* = 0.0004), *I*^2^ = 82%, test for overall effect *Z* = 3.00 (Fig. 5c).

### Correlation between ADC and blood inflammatory markers

**Fig. 6:**
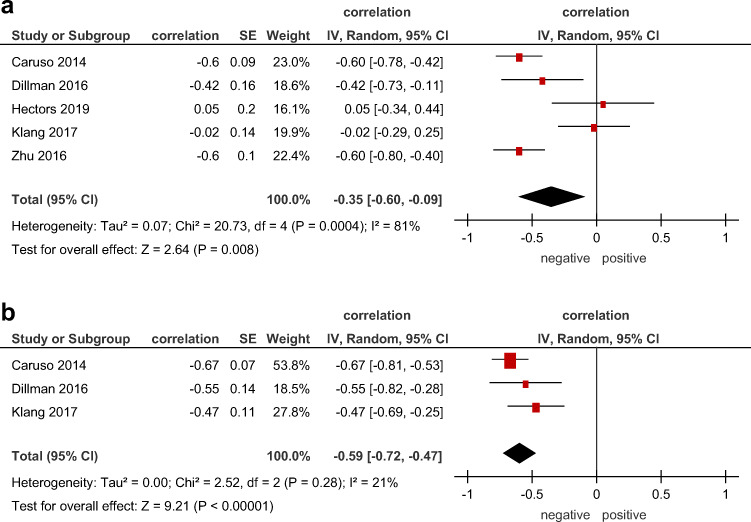
Forest plots of correlation coefficients between (**a**) ADC and C-reactive protein, (**b**) ADC and calprotectin

Associations between ADC and CRP were shown in 5 studies with a total number of 216 patients and represented a weak pooled correlation—0.35 (95% CI = [−0.60, −0.09]), *p* = 0.008, heterogeneity *τ*^2^ = 0.07 (*p* = 0.0004), *I*^2^ = 81%, test for overall effect *Z* = 2.64 (Fig. 6a).

Association between ADC and FCP was reported in 3 studies (139 patients). The pooled correlation coefficient was 0.59 (95% CI = [−0.72, −0.47]), *p* < 0.00001, heterogeneity *τ*^2^ = 0.00 (*p* = 0.28), *I*^2^ = 21%, test for overall effect *Z* = 9.21 (Fig. 6b).

### Diagnostic accuracy of ADC values

The diagnostic value of ADC values was reported in 15 studies. The overall pooled sensitivity to discriminate between involved and non-involved bowel segments was 0.89, the specificity was 0.81, and the AUC was 0.89

For studies only investigating the discriminatory power between no/mild fibrosis to moderate/strong fibrosis, the AUC was 0.84, whereas for studies investigating only acute inflammation, the AUC was 0.91.

## Discussion

The present meta-analysis showed inverse associations between ADC values and disease activity scores in patients with Crohn’s disease. No strong correlation was found for the extent of bowel affection. MRE is performed routinely for most patients with CD due to its excellent diagnostic accuracy. In recent years DWI has become increasingly important in the assessment of bowel inflammation and may complement or potentially replace contrast-enhanced sequences [10]. Our results show that ADC measurements can be applied for disease monitoring in CD. To the best of our knowledge, this is the first comprehensive meta-analysis assessing the correlation of ADC with disease activity parameters in CD. ADC values could therefore potentially be employed as an imaging biomarker to guide treatment decisions. However, there is a clear need for proven threshold values and DWI method standardization.

A strong inverse correlation (*ρ* = −0.80) was observed in the correlation between ADC and CDAI. This finding may be significant in clinical practice. CDAI is used as a gold standard for the clinical evaluation of patients with CD. However, its reproducibility may be limited due to significant inter-observer error, even when performed by experienced physicians [45]. The strong association between ADC and CDAI could be a complement or even an alternative to symptom-guided evaluation. Our results can be considered robust as the total number of patients in the analyzed studies (*n* = 95) was large and reported results were standardized by age groups.

We also found a significant association between ADC and MaRIA score (*ρ* = −0.66). Strong associations were identified in all papers except for one work by Hectors et al [29], in which the long acquisition time of 9 min can be considered unfeasible. The prospective study by Straksyte et al [18], with a large number of patients (*n* = 229), showed a strong inverse correlation between ADC and MaRIA and Clermont indices. Considering the results of the cumulative correlation index as well as the prospective data, ADC measurements may have a strong potential for clinical practice and may be more easily reproduced than the MaRIA score.

We identified a strong correlation between ADC values and SES-CD (*ρ* = −0.66). This indicates the potential of ADC in assessing bowel inflammation. Our results are in line with the study by Buisson et al [33], showing a correlation between ADC and the depth and size of inflammatory ulcerations. The evaluation of inflammatory and fibrotic changes plays a crucial role in CD treatment [21]. Bowel fibrosis is one of the main causes of hospitalization and surgical resection in CD patients [26]. In the last years, a number of studies have been published investigating possible ways to assess and differentiate inflammatory changes from fibrotic histological alterations in bowel walls in patients with CD [20, 21, 24, 26, 28, 32]. Li et al [24] have reported that fibrotic and non-fibrotic bowel wall alterations could be differentiated by means of ADC. Also, mild inflammatory changes could be distinguished from severe ones. However, the ability of ADC to evaluate bowel fibrosis seems to decrease with increasing degrees of bowel inflammation [26].

Previously published studies reported a weak correlation between ADC values and length and thickness of bowel wall inflammation [16, 29]. Our analysis confirmed these results. Shortcomings of the available data, however, must be considered. First, only children were investigated in the included studies. Inflammatory bowel wall changes in children are not associated with fibrosis or fat accumulation, unlike in the adult population. In addition, no standardized measurement of the bowel length and thickness exists, particularly when bowel loops have a complex geometrical form or when bowel peristaltic is not sufficiently suppressed. Standardization of all images of different patients with many causes of wall thickening, like edema, fibrosis, or fat accumulation, or with different bowel distention or peristaltic suppression is challenging [46, 47]. Therefore, the correlation between these parameters and ADC may not be considered reproducible and reliable.

Regarding laboratory data, our findings also support previously published studies, in which Caruso et al [20], Dillman et al [16], and Zhu et al [31] each reported a weak inverse correlation between ADC and CRP. It remains unclear which inflammatory tissue alterations have the strongest impact on diffusion restriction. Zhu et al [31] hypothesized increased cell density in the bowel wall due to influx of lymphocytes, cell swelling, and increased viscosity due to granulomas and micro-abscess. All these processes also lead to a rise in CRP levels. The weak correlation indicates that ADC reduction allows the assessment of local inflammatory changes in the bowel but not of the systematic response, which is reflected by CRP. Thus, both parameters likely reflect distinctive aspects of disease activity.

Our results showed an inverse correlation between ADC and FCP (−0.59), confirming results reported by Dillman et al [16] and Klang et al [22]. FCP increases with inflammatory activity due to neutrophil migration to the gastrointestinal tract and is therefore a common marker of gut inflammation [48]. Restricted diffusion as expressed by ADC in combination with FCP may therefore improve disease monitoring, detect early subclinical inflammatory processes, and lead to better patient outcomes.

One outlier of the present analysis was the study by Hectors et al [29], which showed negative results for clinical parameters. One reason for this could be the employed IVIM technique in the study. The authors reported promising results for the differentiation between normal and abnormal bowel for IVIM-DWI parameters, being superior to ADC values alone. More data are needed to elucidate the potential of the IVIM-DWI technique.

The present results can lead to the hypothesis that ADC values can be used as a valuable imaging biomarker to assess disease severity, presumably better than morphological imaging. ADC measurements may serve as a diagnostic cornerstone for treatment decisions side by side with established clinical parameters like serological inflammation markers.

Our meta-analysis has some limitations. First, many of the included studies were retrospective in nature. Second, it was not possible to standardize the different age groups throughout, and as a result, the heterogeneity was substantial. Third, the acquired data was obtained on different MRI scanners with different technical parameters (magnetic strength, *b*-values, and acquisition time). In addition, the patients’ preparation was not standardized. However, this reflects clinical routine with resulting heterogeneity. Unfortunately, we could not address this by further sub-analyses due to the small number of patients involved in the studies and were thus unable to perform a meta-regression analysis. Fourth, the reference standard to assess inflammation was different throughout the studies. Some used surgical specimens for inflammation, whereas others used endoscopic evaluation. Fifth, this systematic review was not filed in a register, which can result in possible bias regarding the data collection. Furthermore, despite many included studies, some of our subgroups have a small number of patients.

In conclusion, our meta-analysis shows that ADC may be a significant tool for CD disease activity, albeit for selective parameters. We identified moderate-to-strong associations between ADC and CDAI, MaRIA, and SES-CD scores. However, the role of ADC in assessing fibrotic changes in the bowel wall is limited. ADC values can reflect acute inflammatory reactions but no systemic inflammation.

## Abbreviations

ADC: Apparent diffusion coefficient
CD: Crohn’s disease
CDAI: Crohn’s disease activity index
CRP: C-reactive protein
DWI-MRE: Diffusion-weighted sequences
FCP: Fecal calprotectin
MaRIA: Magnetic resonance index of activity
MRE: Magnetic resonance enterography
SES-CD: Endoscopic activity score

## Declarations

### Guarantor

The scientific guarantor of this publication is Prof. Alexey Surov.

### Statistics and biometry

One of the authors has significant statistical expertise.

### Methodology

• retrospective

• meta-analysis
